# Preanalytical considerations in quantifying circulating miRNAs that predict end-stage kidney disease in diabetes

**DOI:** 10.1172/jci.insight.174153

**Published:** 2024-06-24

**Authors:** Eiichiro Satake, Bozena Krolewski, Hiroki Kobayashi, Zaipul I. Md Dom, Joseph Ricca, Jonathan M. Wilson, Dave S.B. Hoon, Kevin L. Duffin, Marcus G. Pezzolesi, Andrzej S. Krolewski

**Affiliations:** 1 Research Division, Joslin Diabetes Center, Boston, Massachusetts, USA.; 2 Department of Medicine, Harvard Medical School, Boston, Massachusetts, USA.; 3 Division of Nephrology, Hypertension, and Endocrinology, Nihon University School of Medicine, Tokyo, Japan.; 4 Eli Lilly and Company, Indianapolis, Indiana, USA.; 5 Department of Translational Molecular Medicine, Saint John’s Cancer Institute, Providence Health and Service, Santa Monica, California, USA.; 6 Department of Internal Medicine, Division of Nephrology and Hypertension, University of Utah School of Medicine, Salt Lake City, Utah, USA.

**Keywords:** Nephrology, Chronic kidney disease, Diabetes, Noncoding RNAs

## Abstract

Our previous study identified 8 risk and 9 protective plasma miRNAs associated with progression to end-stage kidney disease (ESKD) in diabetes. This study aimed to elucidate preanalytical factors that influence the quantification of circulating miRNAs. Using the EdgeSeq platform, which quantifies 2,002 miRNAs in plasma, including ESKD-associated miRNAs, we compared miRNA profiles in whole plasma versus miRNA profiles in RNA extracted from the same plasma specimens. Less than half of the miRNAs were detected in standard RNA extraction from plasma. Detection of individual and concentrations of miRNAs were much lower when RNA extracted from plasma was quantified by RNA sequencing (RNA-Seq) or quantitative reverse transcription PCR (qRT-PCR) platforms compared with EdgeSeq. Plasma profiles of miRNAs determined by the EdgeSeq platform had excellent reproducibility in assessment and had no variation with age, sex, hemoglobin A1c, BMI, and cryostorage time. The risk ESKD-associated miRNAs were detected and measured accurately only in whole plasma and using the EdgeSeq platform. Protective ESKD-associated miRNAs were detected by all platforms except qRT-PCR; however, correlations among concentrations obtained with different platforms were weak or nonexistent. In conclusion, preanalytical factors have a profound effect on detection and quantification of circulating miRNAs in ESKD in diabetes. Quantification of miRNAs in whole plasma and using the EdgeSeq platform may be the preferable method to study profiles of circulating cell-free miRNAs associated with ESKD and possibly other diseases.

## Abstract 2

Compared to traditional platforms, the EdgeSeq platform appears superior for the detection and quantification of end-stage renal disease-associated miRNAs in whole plasma.

## Introduction

miRNAs are short noncoding RNA molecules that can regulate gene expression and protein synthesis both in the cells that produce them and, potentially, in distant cells acting via secretion into circulation (1). Over 2,600 human miRNAs are known (2), and their up- and downregulation result in alterations in translation of many proteins (3–5). Indeed, miRNAs target and regulate approximately 60% of human protein-coding genes (6). In addition to their substantial intracellular functions, most miRNAs are also found in circulation as circulating cell-free miRNA (cfmiRNA) or encapsulated in exosomes. Although the biological functions of circulating cfmiRNAs are still unclear, they can be candidate biomarkers or causal factors involved in the pathogenesis of various diseases (1, 7). Our previous study examined 2,083 cfmiRNAs in individuals with diabetic kidney disease (DKD) using a high-throughput HTG Molecular Diagnostics EdgeSeq direct miRNA platform and identified 17 novel miRNAs associated with declining kidney function and progression to end-stage kidney disease (ESKD) (8). These ESKD-associated miRNAs were detected in whole plasma and comprised 8 risk and 9 protective miRNAs. Several other cfmiRNAs associated with DKD and declining kidney function have been reported (9–11). The findings to date are discrepant and require confirmation in additional studies and validation in additional cohorts. However, before such studies are conducted, preanalytical issues that affect the detectability and accuracy of quantification of cfmiRNAs must be established so that meaningful comparisons can be made.

In the current study, we focused on 3 preanalytical issues that may affect the quantification of cfmiRNAs in plasma. The first issue is variability in detectability and concentration of cfmiRNAs in whole plasma without prior RNA extraction versus miRNAs in RNA extracted from plasma. The second issue is variation in detectability and concentration of miRNAs resulting from application of different platforms for quantification of miRNAs, such as RNA sequencing (RNA-Seq), quantitative reverse transcription PCR (qRT-PCR), and HTG EdgeSeq. Whereas the first 2 platforms require prior extraction of RNA from plasma, the latter does not and allows for cfmiRNA profiling in whole plasma. Many published reports have used these different platforms; however, no major published study has compared results obtained across each of these platforms. The third issue is the impact of clinical confounding factors on miRNA concentration in plasma. Several studies have shown the impact of clinical phenotypes such as age, sex, BMI, and hemoglobin A1c (HbA1c) level on concentrations of cfmiRNAs or in specific tissues (12–20). However, the findings are inconsistent. Extraction of miRNA from plasma is in general not a robust, efficient process, so miRNAs expressed at low levels are often lost. The EdgeSeq direct assay without extraction of miRNA from plasma overcomes this process. Quantification of cfmiRNAs in these studies was determined by different platforms and different biospecimens were used. Our current study focused on the 3 preanalytical issues outlined above and investigated their impact on detectability and quantification of cfmiRNAs in plasma, with a particular focus on our previously reported 17 ESKD-associated miRNAs (8). Very few published reports have attempted to address these questions (21–23), and most importantly, none have examined these in the context of ESKD in diabetes.

## Results

### Profiling of miRNAs in whole plasma and in RNA extracted from plasma.

Detection and quantification of cfmiRNAs vary according to platforms used to measure miRNAs and whether measurements are performed in whole plasma or in RNA extracted from plasma. To study the performance of these platforms, plasma specimens from 4 panels of individuals with diabetes were used. Table 1 shows the clinical characteristics of individuals included in these panels. Overall, 4 platforms were used to measure concentration of cfmiRNAs in whole plasma and in RNA extracted from plasma. As shown in Table 2, the HTG EdgeSeq platform detected 2,002 out of the 2,083 miRNAs measured on this platform in whole plasma. This number of cfmiRNAs and their concentrations will be considered reference values in this report. In RNA extracted from plasma, the same platform detected only 930 miRNAs (46% of all reference miRNAs); 1,072 plasma miRNAs were no longer detectable following RNA extraction. In the same RNA, the RNA-Seq using 2 different small RNA (sRNA) sample preparation kits (RNASeq_1 and RNASeq_2) detected 13% and 22% of all reference miRNAs. A slightly higher proportion (27%) of reference miRNAs was detected by qRT-PCR in RNA extracted from pooled plasma specimens.

**Figure 1:**
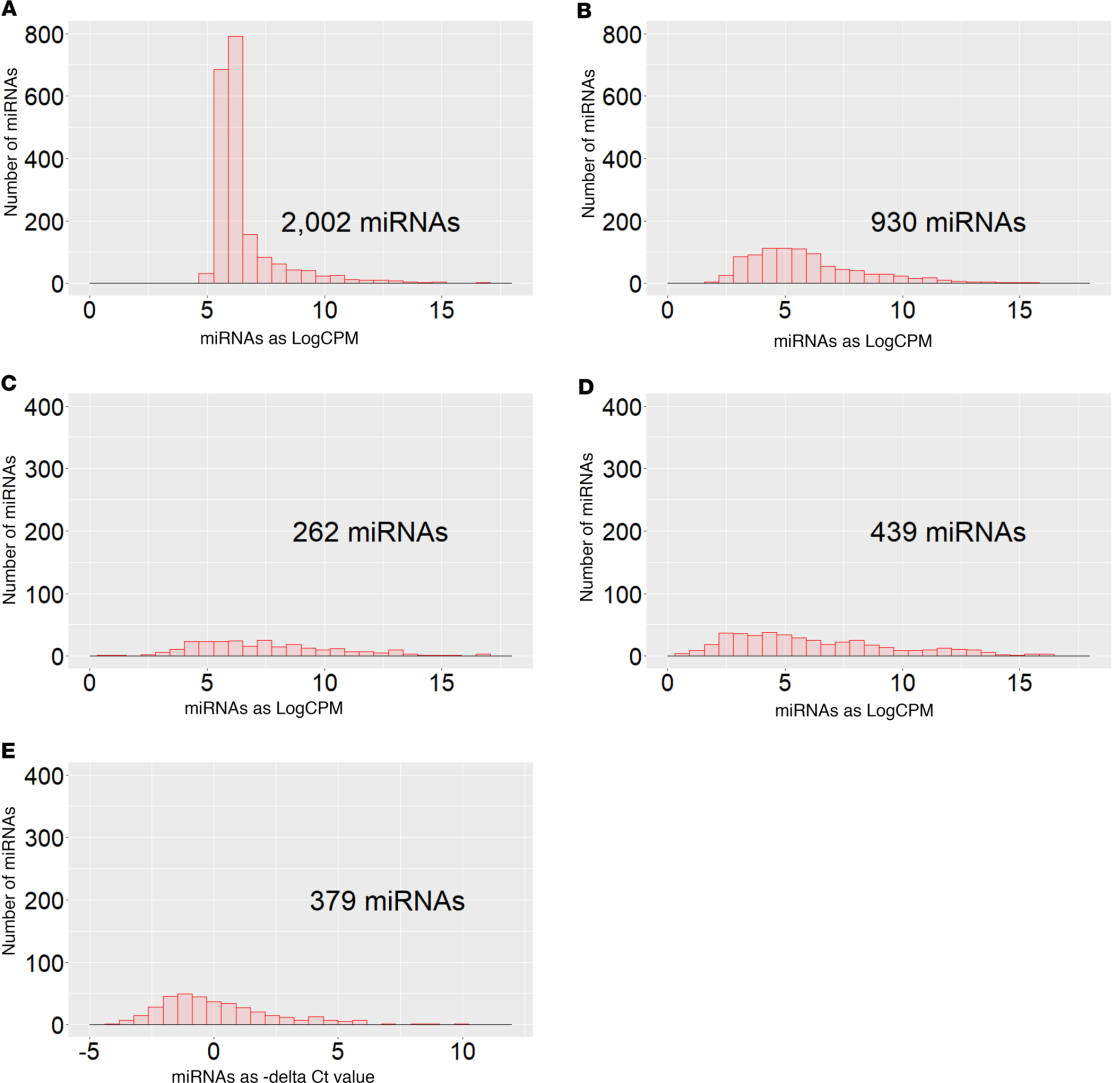
Distributions of number of detected cfmiRNAs according to raw read count of miRNAs in study panels, according to used platforms for quantification of cfmiRNAs. (**A**) cfmiRNAs in plasma quantified by the HTG EdgeSeq platform. (**B**) cfmiRNAs in RNA extracted from plasma quantified by EdgeSeq. (**C**) cfmiRNAs in RNA extracted from plasma and quantified by RNASeq_1 (SeqMatic). (**D**) cfmiRNAs in RNA extracted from plasma and quantified by RNASeq_2 (LC Sciences). (**E**) miRNAs in RNA extracted from pooled plasma and quantified by qRT-PCR. The cfmiRNAs were considered detectable if they had concentrations of more than 1 CPM in more than 80% of the samples (7 or more samples out of 8) in EdgeSeq or in more than 50% of the samples in RNA-Seq or Ct values less than 30 in qRT-PCR.

Figure 1 shows distributions of detected miRNAs according to their concentration determined by the different platforms in whole plasma and in RNA extracted from plasma and RNA-Seq library sample preparation kit. Supplemental Table 1 (supplemental material available online with this article; https://doi.org/10.1172/jci.insight.174153DS1) provides results, detectability, and concentration, for each of the reference miRNAs measured by these different platforms and kits.

**Figure 2:**
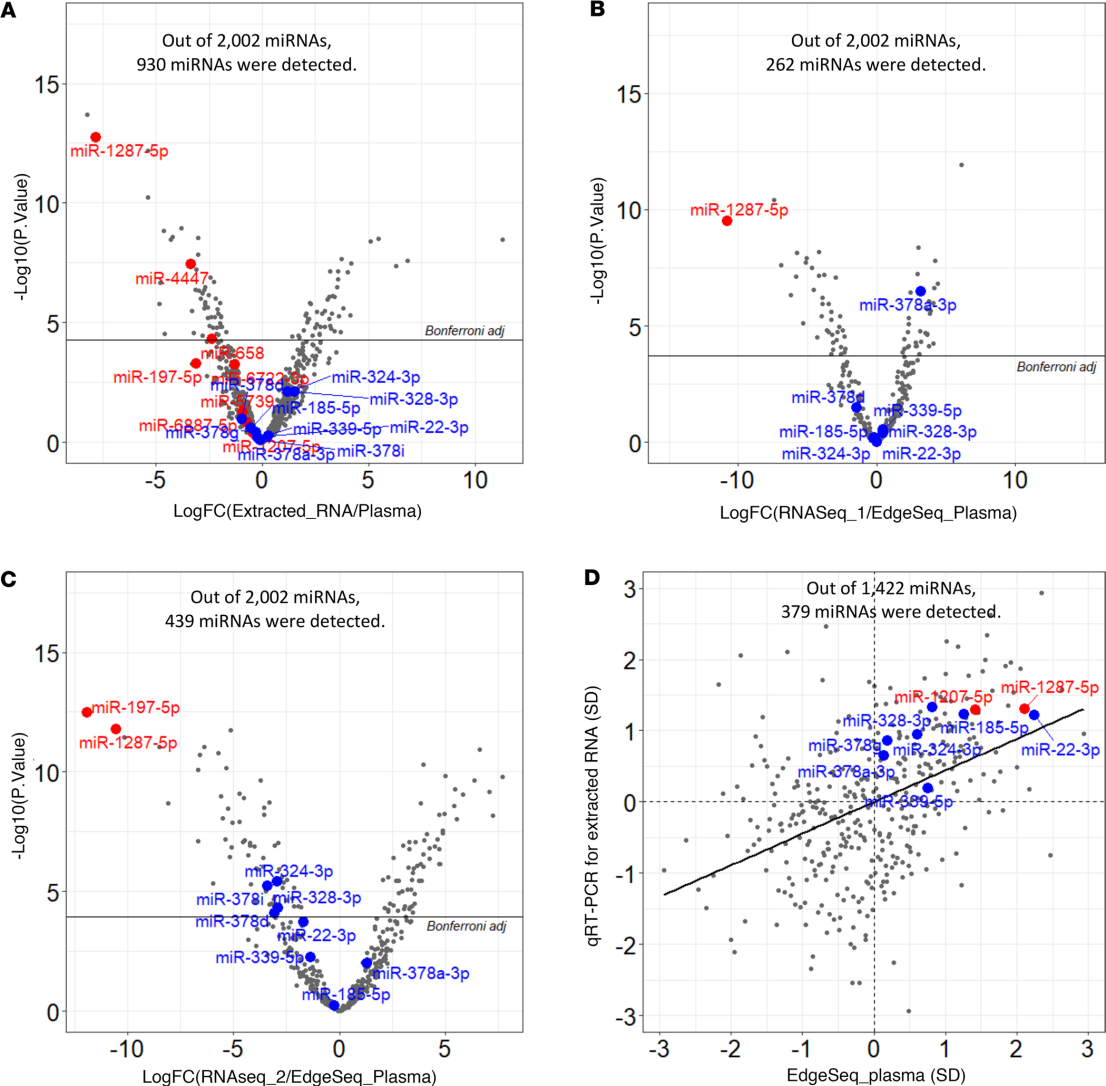
Fold-changes of concentration of cfmiRNAs in extracted RNA from plasma over concentration of cfmiRNAs in whole plasma, according to different platforms used. (**A**) RNA extracted from plasma over concentration of miRNAs in whole plasma measured by EdgeSeq, (**B**) miRNAs in RNA extracted from plasma measured by RNA-Seq (SeqMatic) over concentration of cfmiRNAs in whole plasma measured by EdgeSeq, and (**C**) cfmiRNAs in RNA extracted from plasma measured by RNA-Seq (LC Sciences) over concentration of cfmiRNAs in whole plasma measured by EdgeSeq analyzed using the voom, lmFit, and eBayes functions in the limma package. FC, fold-change. (**D**) Spearman correlation coefficients between concentration of cfmiRNAs in RNA extracted from plasma measured by qRT-PCR and concentration of miRNAs in whole plasma measured by EdgeSeq. Red dots indicate risk ESKD-associated cfmiRNAs. Blue dots indicate protective ESKD-associated miRNAs. Detailed comparisons are shown in Table 3.

Figure 2 compares the concentration of miRNAs detected in RNA extracted from plasma measured by different platforms and different RNA-Seq library preparation kits over concentration of miRNAs in whole plasma measured by EdgeSeq. Figure 2A shows a volcano plot of FCs of the concentrations of cfmiRNAs in RNA extracted from plasma compared with concentrations of miRNAs in whole plasma measured using the EdgeSeq platform. Concentrations of miRNAs measured in the 2 specimens were different. Out of 930 miRNAs that were detected in both specimens, 53 (5.7%) miRNAs had significantly lower concentrations (i.e., lower FC) in extracted RNA than in whole plasma, and 46 (4.9%) miRNAs had concentrations significantly higher (i.e., higher FC) in extracted RNA than in whole plasma. A lower number of cfmiRNAs were detected and more discrepant concentrations were found when cfmiRNAs in RNAs extracted from plasma were measured by RNASeq_1 and RNASeq_2 (Figure 2, B and C). Figure 2D shows a comparison of cfmiRNA concentrations in whole plasma measured by the EdgeSeq platform (*N* = 8) and in extracted RNAs from pooled plasma measured by qRT-PCR (*N* = 40). Whereas only 379 cfmiRNAs were detected using both platforms, substantial discrepancies in their concentration were observed.

### Profiling of ESKD-associated miRNAs in whole plasma versus in RNA extracted from plasma.

Table 3 compares concentrations of the 17 ESKD-associated cfmiRNAs determined by different platforms in whole plasma and in RNA extracted from plasma and different RNA-Seq library preparation kits. Among risk cfmiRNAs, the EdgeSeq platform detected all 8 risk miRNAs in RNA extracted from plasma; however, their concentrations were significantly lower than in whole plasma. These miRNAs were hardly detected by the RNA-Seq and qRT-PCR platforms. In striking contrast, among the 9 protective miRNAs, 6 of them (miR-339-5p, miR-324-3p, miR-185-5p, miR-22-3p, miR-378a-3p, and miR-328-3p) were detected by HTG EdgeSeq and RNA-Seq platforms, and they had similar concentrations independent of whether whole plasma or RNA extracted from plasma was used. qRT-PCR produced very discrepant results relative to the other examined platforms.

The concentrations of 17 ESKD-associated miRNAs determined by each platform in RNAs extracted from plasma and RNA-Seq library kit were correlated with concentrations of these miRNAs in whole plasma determined by EdgeSeq platform (i.e., reference concentrations). The results are shown in Supplemental Table 2. The concentration of risk miRNAs determined in whole plasma did not correlate with the concentration of these miRNAs determined in RNA extracted from plasma. On the other hand, the concentration of protective cfmiRNAs determined in whole plasma correlated with the concentration of some of the cfmiRNAs determined in RNA extracted from plasma. However, correlation coefficients varied widely, from no correlation to strong correlation, likely due to the small number of individuals (*n* = 8) used in this analysis.

### Stability of miRNA level in whole plasma according to duration of storage.

**Figure 3:**
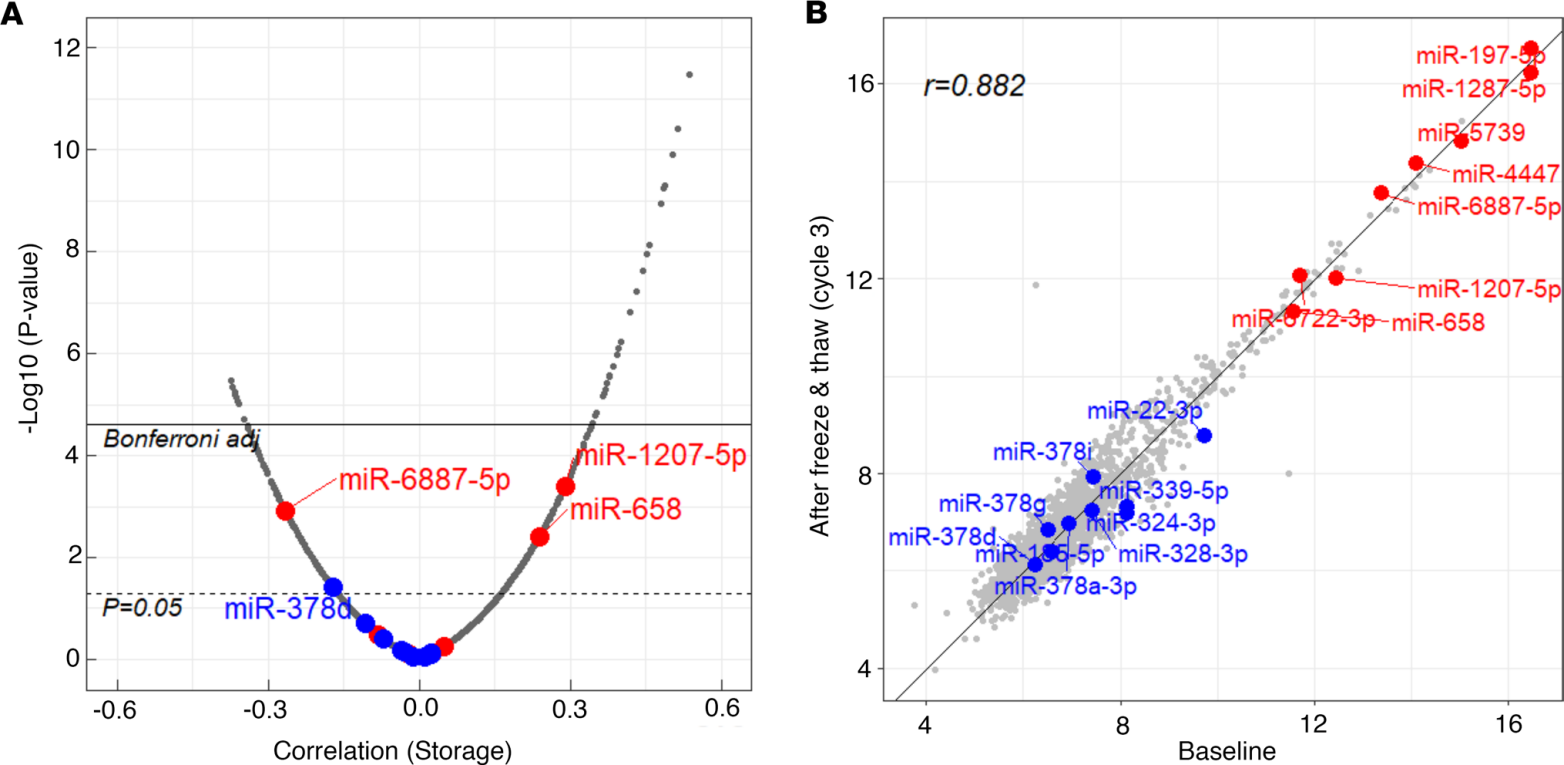
Stability of miRNAs in plasma and duration of storage and freeze/thaw cycles. (**A**) Volcano plot of Spearman correlation coefficients between miRNA concentrations in whole plasma and duration of plasma storage in individuals in panel 3 (*N* = 145). Red dots indicate risk ESKD-associated miRNAs, and blue dots indicate protective ESKD-associated miRNAs. The mean ± SD of storage duration (years) was 9.3 ± 2.3 years. (**B**) Comparison of plasma miRNA concentrations between baseline and after 3 cycles of freeze and thaw using Spearman correlation.

The duration of storage of archived specimens used in research could affect the stability of cfmiRNAs and their observed association with various diseases. To assess this, we examined the correlation between the concentration of cfmiRNAs in plasma and variable storage time (ranging from 4 to 14 years) of these specimens at –80°C. The Spearman correlation coefficients are shown in Figure 3A as a volcano plot. Only a small number of miRNAs showed significant (after Bonferroni adjustment) positive correlations with duration of storage. Among ESKD-associated cfmiRNAs, 3 (miR-6887-5p, miR-658, and miR-1207-5p) showed nominal correlation with increasing duration of storage, though these were not statistically significant (Supplemental Table 3). We also assessed stability of miRNAs after freeze/thaw cycles (Figure 3B), which indicates that most miRNAs were stable even after multiple cycles.

### Changes in concentrations of miRNAs in whole plasma of individuals during follow-up observations.

**Figure 4:**
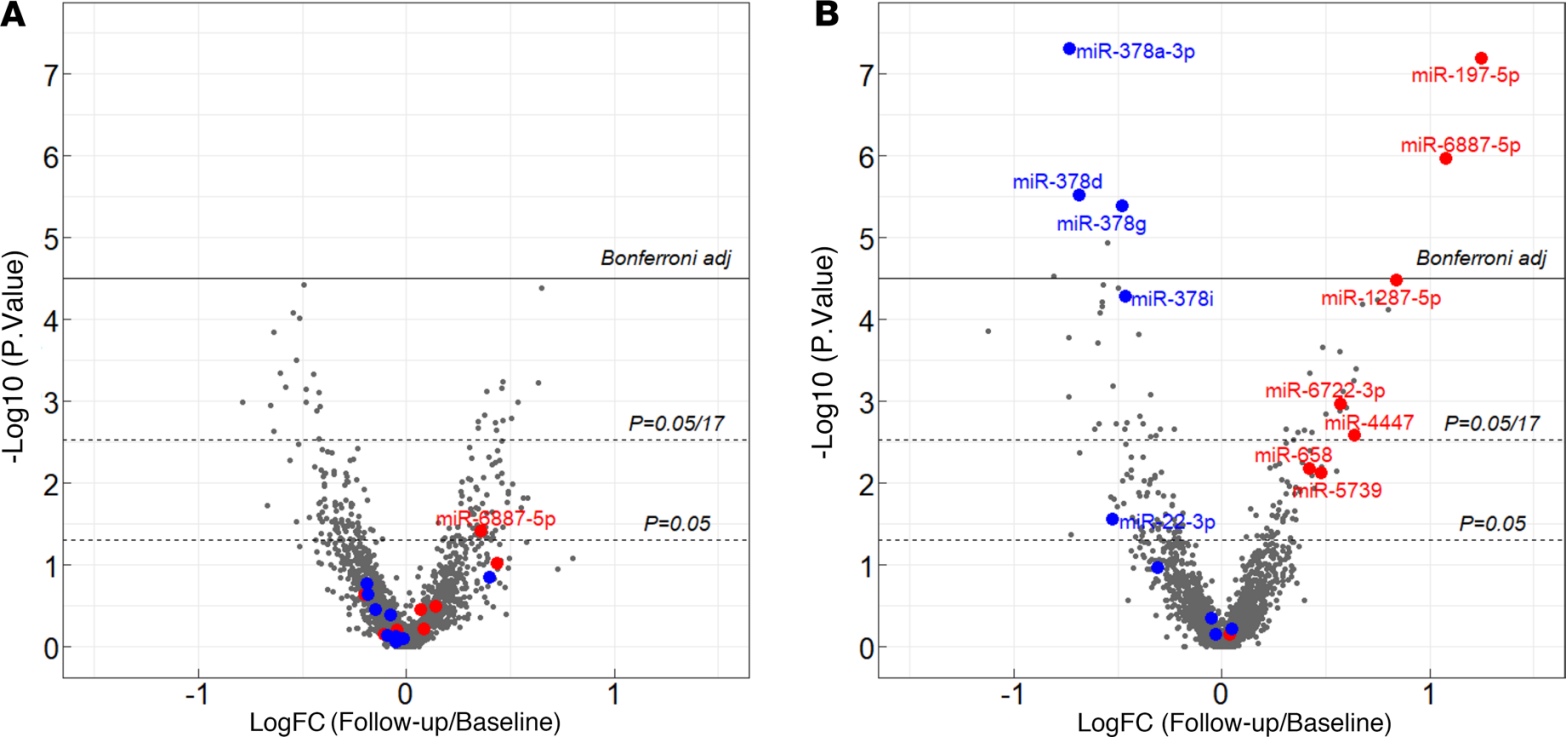
FCs of concentration of cfmiRNAs measured with EdgeSeq platform in whole plasma obtained at follow-up over concentration of miRNAs in whole plasma obtained at baseline, from individuals in panel 4 (*N* = 96). (**A**) Results obtained for 52 T1D individuals who did not progress to ESKD during 7–15 years of follow-up. Median (25th % and 75th %) of duration between baseline and follow-up measurements was 3.7 (2.2, 5.3) years. (**B**) Results obtained for 44 T1D individuals who progressed to ESKD during 7–15 years of follow-up. Median (25th % and 75th %) of duration between baseline and follow-up measurements was 4.1 (2.5, 6.3) years. Red dots indicate risk miRNAs and blue dots indicate protective cfmiRNAs. A paired nonparametric analysis using the Wilcoxon rank sum test was used for comparison.

Figure 4 shows comparisons of concentrations of cfmiRNAs in plasma obtained at baseline and follow-up examinations in individuals with T1D. The mean duration between baseline and follow-up visits was 4.3 years. Surprisingly, no significant change in the concentration of plasma cfmiRNAs was observed in individuals who did not progress to ESKD during follow-up (Figure 4A). In contrast, in individuals who progressed to ESKD, plasma concentration of 5 risk ESKD-associated cfmiRNAs (miR-197-5p, miR-6887-5p, miR-1287-5p, miR-6722-3p, miR-4447) significantly increased, and concentration of 4 protective ESKD-associated cfmiRNAs (miR-378a-3p, miR-378d, miR-378g, and miR-378i) significantly decreased during follow-up (Figure 4B). Of note, no other reference cfmiRNAs significantly increased or decreased during follow-up among individuals who progressed to ESKD.

### Clinical characteristics and whole-plasma concentration of miRNAs.

**Figure 5:**
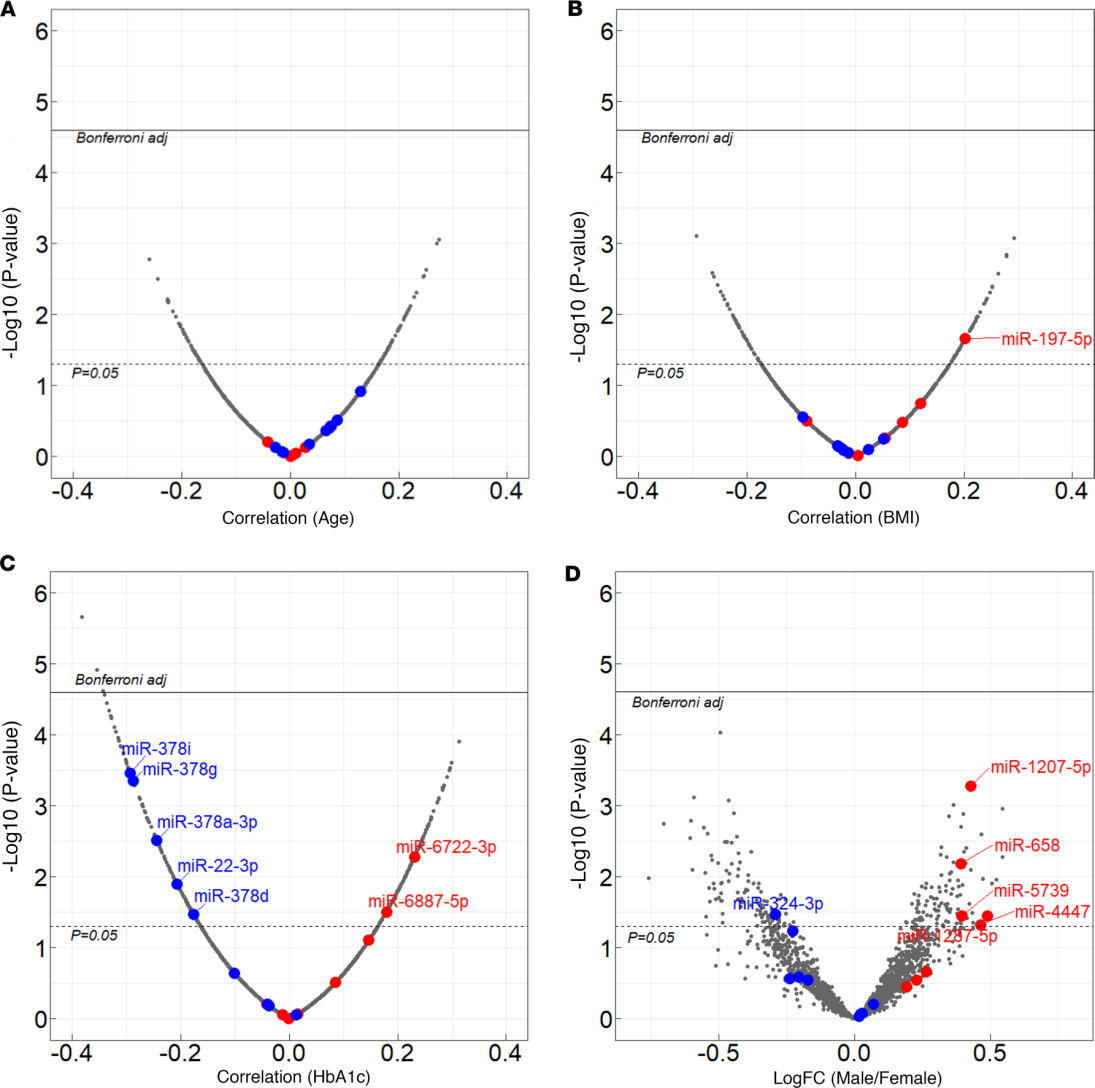
Relationship among demographic/clinical variables and concentration of cfmiRNAs in plasma measured with EdgeSeq platform in 145 T2D individuals in panel 3. Red dots indicate risk ESKD-associated cfmiRNAs and blue dots indicate protective ESKD-associated miRNAs. (**A**–**C**) Volcano plots for Spearman correlation coefficients between cfmiRNA concentrations in plasma and age, HbA1c (*N* = 145), and BMI (*N* = 128). (**D**) Volcano plots for FCs in plasma concentration of cfmiRNAs in men over plasma concentration of miRNAs in women. Data were analyzed using the voom, lmFit, and eBayes functions in the limma package.

Associations between plasma cfmiRNA profiles and disease risk may be influenced by demographic and/or clinical factors. Figure 5, A–C, show volcano plots for Spearman correlation coefficients between concentration of 2,002 miRNAs in whole plasma and 3 clinical characteristics that are important prognosis factors of ESKD (age, BMI, and HbA1c). After adjustment for multiple comparisons, none of these characteristics was associated with concentrations of cfmiRNAs in plasma, including the 17 ESKD-associated cfmiRNAs (Supplemental Table 4). Similarly, concentrations of cfmiRNAs were not different between men and women (Figure 5D).

## Discussion

This study examined preanalytical factors that may affect quantification of cfmiRNAs in plasma in individuals with diabetes. We discovered that measuring miRNAs in whole plasma without prior RNA extraction versus RNA extracted from plasma, the platform used for quantification, and the sRNA library preparation kit all had profound impacts on the detectability and accuracy of miRNA determinations. In contrast, long-term storage of plasma at –80°C had minimal impact on concentration of cfmiRNAs. Similarly, clinical variables, such as age, sex, BMI, and HbA1c, were not associated with concentration of cfmiRNAs in plasma. These findings highlight challenges and considerations in validating findings across studies, including our findings and their implementation in the prediction of risk of ESKD, where sample preparation methods, miRNA quantification platforms, sRNA library preparation, and potential confounders may differ.

Because the EdgeSeq platform quantifies miRNAs in biofluids both with and without prior extraction of RNA, we were able to compare miRNA profiles in whole plasma (a reference) and in RNA extracted from plasma. Significant differences were found between the 2 isolation approaches. First, RNA extraction reduced the number of detectable cfmiRNAs by 46%, and second, concentrations of many detectable cfmiRNAs in RNA extracted from plasma were different from those in whole plasma. Several factors may account for loss of cfmiRNAs in RNA extracted from plasma. In whole plasma, miRNAs are generally found within extracellular vesicles, such as exosomes, or associated with RNA-binding proteins and lipoproteins such as Argonaute-2 or HDL-cholesterol (24–26). These miRNAs are protected from degradation by RNases found in plasma, and their concentrations are stable for long periods, as shown in this study. A large proportion of reference miRNAs were not detected in extracted RNA from plasma, most likely due to their degradation or, perhaps, their selective removal during RNA extraction. TRIzol, or similar reagents used for RNA extraction, for example, may directly degrade miRNAs or their binding proteins and, thereby, lead to their degradation (27). The RNA extraction procedure logistics has a greater loss of low-level cfmiRNA recovery, whereas the direct assay allows better recovery. Regarding the selective removal of miRNAs during RNA extraction, Kim et al. previously reported that miRNAs with low guanine-cytosine content (i.e., GC content) are lost during RNA extraction (27). Also the direct extraction assay of plasma picks up exosomes containing miRNA. Our research supports that observation (Supplemental Figure 1).

The detectability and concentration of cfmiRNAs determined in RNA extracted from plasma varied significantly across miRNA profiling platforms and sRNA library preparation kits. In this study, 3 platforms, EdgeSeq and RNA-Seq with 2 sRNA sample preparation kits (SeqMatic’s TailorMix miRNA Sample Preparation Kit V2, RNASeq_1; and Illumina’s TruSeq Small RNA Sample Prep Kit, RNASeq_2), were compared. sRNA libraries prepared using the TailorMix kit detected 262 (28%) and sRNA libraries prepared using the TruSeq kit detected 439 (47%) out of 930 reference miRNAs detected by EdgeSeq in plasma that was first subjected to RNA extraction.

The large discrepancies in detectability and quantification of cfmiRNA among these platforms can be accounted for by multiple factors, including 1) technical bias such as different protocols and kits for RNA preparation, 2) adapter ligation bias, 3) use of different T4 RNA ligases, and 4) PCR bias during cDNA amplification, resulting from differing PCR efficiencies while amplifying molecules of different length and secondary structure (28). The qRT-PCR platform, considered the gold standard for mRNA/gene expression profiling, has also been used to quantify cfmiRNAs. Unfortunately, in this study, this platform was the least effective in detecting ESKD-associated cfmiRNAs. Although mechanisms responsible for these different results can be studied further, the 3 platforms are not suitable for detection and quantification of cfmiRNAs associated with ESKD.

EdgeSeq is a next-generation sequencing–based miRNA profiling platform that measures miRNAs in whole plasma, including exosomal and protein/lipid-bound cfmiRNAs. Only approximately 15 μL of plasma is needed to measure 2,083 candidate mature miRNAs. Other cfmiRNA extraction assays described above require in general several milliliters of plasma. The samples are run on the HTG EdgeSeq processor using the EdgeSeq miRNA Whole Transcriptome Assay, in which an excess of nuclease protection probes complementary to each miRNA hybridize to their target. Libraries are sequenced on a next-generation high-throughput sequencer, such as a NextSeq 550 (Illumina), for quantification. Our study detected 2,002 out of 2,083, i.e., 96%, of all miRNAs on this platform. Measurement of the concentrations of these cfmiRNAs was extremely highly reproducible. Furthermore, the concentrations of almost all miRNAs were similar when measured in baseline and follow-up samples of whole plasma collected approximately 4 years apart from a large group of individuals. It is important to note that in individuals who progressed to ESKD during 7–15 years of follow-up, substantial changes in miRNA concentrations occurred almost exclusively in ESKD-associated miRNAs; i.e., the concentrations of 4 protective cfmiRNAs decreased during follow-up, and the concentrations of 5 risk ESKD-associated cfmiRNAs increased during follow-up. These findings imply that ESKD-associated miRNAs changed with declining kidney function and progression to ESKD.

We found no significant correlation between the concentrations of cfmiRNAs in whole plasma and clinical variables/confounders such as age, BMI, HbA1c, and sex. These findings conflict with previous reports that showed modest association of very specific cfmiRNAs with some clinical characteristics (13–16). However, as reviewed in Supplemental Table 5, the reported findings were inconsistent; i.e., there were no specific cfmiRNAs associated with clinical variables that were replicated among those studies. Importantly, the comparison of our findings with those in the prior studies is difficult because those studies profiled cfmiRNAs following RNA extraction and included variable biospecimens and various platforms for quantification of circulating miRNAs. Our study found many miRNAs associated with clinical variables at nominal significance; however, the findings became statistically insignificant when appropriate adjustments for multiple comparisons were applied. Importantly, the results reported in most of the previous publications (summarized in Supplemental Table 5) were not adjusted for multiple comparisons.

Interestingly, all ESKD-associated cfmiRNAs were detected in whole plasma as in RNA extracted from plasma using the EdgeSeq platform. Concentrations of the risk cfmiRNAs detected in RNA extracted from plasma, however, were substantially lower than those in whole plasma; in contrast, concentrations of protective miRNAs were similar irrespective of whether RNA extraction was used. Similar patterns were seen when cfmiRNAs were quantified using RNA-Seq or qRT-PCR platforms. One noticeable difference was that the risk ESKD cfmiRNAs were not detectable at all in RNA extracted from plasma. The above findings provide evidence that the ESKD-associated risk cfmiRNAs are “lost” not only during extraction of RNA from plasma but also during sRNA library processing. This vulnerability is not seen for ESKD-associated protective cfmiRNAs, which may reflect ligation bias, GC contents of miRNAs, and primary sequence or secondary structure difference between risk and protective ESKD-associated miRNAs. Another potential reason is a difference in the miRNA carrier between risk and protective cfmiRNAs. cfmiRNAs are found in free form and can be carried in blood by 3 forms: 1) extracellular vesicles (e.g., exosome), 2) Argonaute proteins (e.g., Ago2), and 3) lipoproteins (e.g., HDL-cholesterol). Arroyo et al. and Turchinovich et al. showed that the majority of nuclease-resistant extracellular miRNAs in plasma are outside exosomes and are bound to the Ago2 protein (24, 29). It is possible that RNA extraction reagent denatures or impacts the Ago2 protein, leading to degradation of risk ESKD-associated miRNAs during the process. Further research is needed to clarify the carriers of ESKD-associated cfmiRNAs and the stabilities of exosomal and protein-bound cfmiRNAs in circulation. This inference is not true for ESKD-associated protective miRNAs. It is possible that the striking differences for the ESKD risk cfmiRNAs versus the protective ones are due to different carriers of these cfmiRNAs. Further work is needed to fully understand this observation as there are no recent publications that could explain these findings.

The discrepant results obtained for the 17 ESKD-associated cfmiRNAs determined in whole plasma versus in RNA extracted from plasma and different concentrations of these cfmiRNAs according to miRNA quantification platforms have important implications for future translational research. At present, the EdgeSeq platform is the only platform to reliably measure concentration of ESKD-associated cfmiRNAs in whole plasma and, therefore, is the only platform that can reliably be used to study the mechanisms through which these cfmiRNAs impact disease processes leading to progressive kidney function decline and ESKD in diabetes. Similarly, this platform is the best to determine cfmiRNAs as good predictors of ESKD.

Finally, some limitations of our study should be considered. The weakness of our study is that plasma specimens from only 8 individuals were used. An increased number of individuals included in this study might improve correlation between protective ESKD-associated cfmiRNAs determined in whole plasma and in RNA extracted from plasma. Furthermore, because we focused on individuals with diabetes who had risk of progression to ESKD, the generalizability of our findings is uncertain regarding other diseases as they would be associated with other specific miRNAs. Additionally, our study assessed cfmiRNA profiles in plasma specimens; as such, it is not clear whether similar results will be found if cfmiRNAs are measured similarly in serum or other biofluids. An issue regarding assessment of serum is clotting of blood allows cells in the clot to release miRNA into the serum, which can be high depending on how long the blood was allowed to clot.

Finally, we evaluated only a single platform, EdgeSeq, which quantifies miRNAs in whole plasma without prior RNA extraction. Similar to EdgeSeq, the Abcam FirePlex source location system measures cfmiRNAs (up to 65 preselected miRNAs) from 10 μL of plasma or serum without requiring RNA purification; however, this platform became unavailable in 2023 (30).

In conclusion, our study demonstrated the important role of preanalytical factors in the detection and quantification of miRNAs in plasma. To accurately measure and evaluate miRNAs, especially our previously reported ESKD-associated miRNAs, utilizing the HTG EdgeSeq platform on plasma samples without RNA extraction is recommended.

## Methods

### Sex as a biological variable.

Our study examined men and women, and similar findings are reported for both sexes.

### Study participants.

Plasma specimens used in this study were obtained from individuals participating in the Joslin Kidney Study (JKS) (31, 32). The JKS is a longitudinal, observational study that investigates the determinants and natural history of kidney function decline in individuals with T1D and T2D. The recruitment, the follow-up examination for the participants, and the study protocols were reported in our previous publications (31, 32). Plasma was obtained from the participants by standard procedure and stored at –80°C until analysis.

Four panels of individuals with available plasma specimens were used in this study (see Table 1). To compare cfmiRNA profiles from plasma with RNA extraction or direct isolation, we randomly selected samples from 8 individuals from the JKS (Panel 1). To profile miRNAs using qRT-PCR, pooled plasma samples from 40 individuals with T1D, normal kidney function, and normal urinary albuminuria were analyzed (Panel 2). To assess the stability of miRNAs following long-term storage, Spearman correlations between plasma concentration of miRNAs and storage duration in 140 individuals with T2D were analyzed (Panel 3). Stability/tracking of concentrations of miRNAs over time was analyzed by comparing changes in plasma cfmiRNA profiles between specimens obtained at baseline and follow-up in individuals who progressed to ESKD during follow-up (*n* = 44) and individuals who did not (*n* = 52) (Panel 4). Correlations between plasma concentration of cfmiRNAs and clinical variables were examined in 145 randomly selected T2D individuals (Panel 3).

### RNA extraction from plasma.

Total RNA was isolated from the selected samples using 180 μL of plasma from each individual and QIAGEN’s miRNeasy Serum/Plasma kit according to the manufacturer’s protocol as previously published (33).

### HTG EdgeSeq assay to measure concentration of miRNAs.

To determine the majority of currently known miRNAs in whole plasma, we used HTG Molecular Diagnostics’ HTG EdgeSeq miRNA sequence platform (8, 17, 21, 22, 34, 35). This platform can measure profiles of 2,083 miRNAs without prior RNA extraction. The process isolates and detects mature circulating cfmiRNAs in plasma, including exosomal and protein/lipid-bound miRNAs. Fifteen-microliter aliquots of plasma were used to perform these measurements. An equal volume of the HTG plasma lysis buffer was added to each sample prior to submitting the samples to HTG Molecular Diagnostics for miRNA profiling. To compare miRNA concentrations between whole plasma assay and extracted RNA, we isolated cfmiRNA from additional aliquots of these same plasma samples using QIAGEN’s miRNeasy Serum/Plasma Kit. miRNAs from these samples were eluted using 14 μL RNase-free water and were submitted to HTG Molecular Diagnostics for analysis using the HTG EdgeSeq platform.

### RNA-Seq to determine miRNAs in RNA extracted from plasma.

RNA-Seq of sRNAs, including miRNAs, was performed by SeqMatic (RNASeq_1) and LC Sciences (RNASeq_2). Briefly, sRNA libraries for the SeqMatic platform were prepared using equal volumes of total RNA from each sample. Total RNA from each sample was used for 5′ and 3′ ligation of Illumina adapters, cDNA synthesis, and PCR amplification using the SeqMatic’s TailorMix miRNA Sample Preparation Kit V2 according to the manufacturer’s recommendations. Purified sRNA libraries were sequenced as 50 bp single-end reads in a single lane using an Illumina HiSeq 2000 DNA sequencer at SeqMatic’s miRNA Sequencing Facility to achieve more than 10 million mappable reads per sample. The resulting sequence data were analyzed using an in-house pipeline that included initial processing and demultiplexing using Illumina’s CASAVA v1.8.2 software pipeline, quality control checks on the raw fastq files using the FastQC software (http://www.bioinformatics.babraham.ac.uk/projects/fastqc), and adapter and quality trimming using the FASTX toolkit (http://hannonlab.cshl.edu/fastx_toolkit/index.html). The preprocessed reads were mapped to the human genome (version hg19) using the STAR aligner (36). FeatureCounts (37) was used to generate summed read count data for miRNAs using miRBase v21 (http://www.mirbase.org/). In total, 2,588 miRNAs were sequenced by the SeqMatic RNA-Seq platform, and among them, 2,082 overlapped with those included on the HTG EdgeSeq platform.

For small RNA-Seq performed by LC Sciences (RNASeq_2), total RNA was extracted by the company from 200 μL of plasma using TRIzol reagent (Invitrogen) following the manufacturer’s procedure. The total RNA quality and quantity were analyzed using Bioanalyzer 2100 (Agilent) with RNA integrity number greater than 7.0. Approximately 1 μg of total RNA was used to prepare sRNA libraries using the commercially available TruSeq Small RNA Sample Prep Kit (Illumina) according to the manufacturer’s protocol. Then 50 bp single-end read sequencing was performed on a HiSeq 2500 at LC Sciences following the vendor’s recommended protocol. Small RNA-Seq data were analyzed using ACGT101-miR (LC Sciences) to remove adapter dimers; low-complexity, common RNA families; and repeats. Unique sequences with length in 18–26 nucleotides were then mapped to specific species precursors in miRBase 22.0 by BLAST search to identify known miRNAs and novel 3p- and 5p-derived miRNAs. In total, 579 miRNAs were detected by the LC Sciences RNA-Seq platform, and among them, 439 overlapped with the HTG EdgeSeq platform.

### qRT-PCR to quantify cfmiRNAs in extracted RNA from plasma.

Plasma specimens from the 40 individuals with T1D and normal urinary albuminuria (considered healthy individuals) were selected from the JKS for this study. Total RNA was isolated from 180 μL plasma from each specimen using the QIAGEN miRNeasy Serum/Plasma Kit. The plasma samples were pooled and then RNA was extracted.

For analyses of 1,805 miRNAs by qRT-PCR, the human miRNome miScript miRNA PCR Array (V16.0, 384-well) including 1,066 assays (QIAGEN) and custom miScript miRNA PCR Array (QIAGEN) including 739 additional miRNAs were used. Reverse transcription of RNA isolated from plasma was performed using the miScript II RT Kit with miScript HiSpec Buffer (QIAGEN). Isolated RNA (1.5 μL) was used to prepare a 10 μL reverse transcription reaction as specified by the manufacturer. Preamplification was performed using miScript PreAMP PCR Kit and a custom miScript PreAMP Primer Mix according to the manufacturer’s protocol. Following the reverse transcription and preamplification of miRNAs included on these arrays, miRNA concentrations were assayed by SYBR Green–based qRT-PCR using 0.25 μL of diluted cDNA in a 10 μL reaction on an Applied Biosystems 7900HT Fast Real-Time PCR System. Amplification results were analyzed using SDS 2.4 software (Applied Biosystems). miRNAs with Ct values 30 were considered undetectable. Global mean normalization, as described by Mestdagh et al., was used to normalize the resulting qRT-PCR data (38). Relative quantification values were calculated using the ΔΔCT method. FCs were calculated using the equation 2^−ΔΔCt^. Among the 1,805 miRNAs, 1,484 overlapped with those included on the HTG EdgeSeq platform.

### Analysis of HTG EdgeSeq data.

miRNAs with low concentrations were filtered out using edgeR R package (Version 3.12.1) (39). We defined detectable concentrations as more than 1 CPM in more than 80% of the individuals in Panel 1 (7 out of 8 samples).

### Data normalization of HTG EdgeSeq and RNA-Seq data.

For data analysis of HTG EdgeSeq and RNA-Seq platforms, we applied quantile normalization for each miRNA read count with sample weights (40) using the edgeR (version 3.42.4) (39) and limma (version 3.56.2) (41) R packages from Bioconductor. We considered miRNAs detectable if they had concentrations of more than 1 CPM in more than 90% of our samples in Panels 2 to 4 in HTG EdgeSeq and 50% of samples in RNA-Seq according to the previous publications (8, 42–44). We then applied quantile normalization, a nonscaling approach that forces the distribution of read counts in all experimental samples to be equivalent and assumes that 1) most target miRNAs are not differentially expressed and 2) the true expression distribution of miRNAs is similar across all samples (37).

### Statistics.

Clinical characteristics are reported as counts and percentages (proportions) for categorical variables, means with standard deviations for normally distributed continuous variables, and medians and quartiles for variables with skewed distributions. The normality of distribution was assessed by the Shapiro-Wilk and Kolmogorov-Smirnov tests. Differences between the 2 groups with nonparametric distribution were tested using the Wilcoxon rank sum test. Comparisons of differences in cfmiRNA concentrations were assessed by FC analysis using the edgeR package (Version 3.42.4) and voom, lmFit, and eBayes functions in the limma package (Version 3.56.2). Correlations between clinical characteristics and cfmiRNA concentrations were estimated by Spearman rank correlation test. To determine differential miRNA expression between baseline and follow-up samples, we applied a paired nonparametric analysis using the Wilcoxon rank sum test by SAS. Bonferroni correction for the number of measured miRNAs was applied. All statistical analyses were performed with SAS version 9.4 and R version 4.0.3. *P* values less than 0.05 were considered statistically significant.

### Study approval.

Study protocols for recruitment and examination of individuals in the JKS and related consent procedures were approved by the Joslin Diabetes Center Institutional Review Board.

### Data availability.

Data are available in the Supporting Data Values XLSX file. The RNA-Seq data included in this study have been deposited in National Center for Biotechnology Gene Expression Omnibus (accession no. GSE266819; https://www.ncbi.nlm.nih.gov/geo/).

## Author contributions

ES designed the study, performed the experiments, analyzed the data, and wrote the manuscript. BK, HK, ZIMD, and JR contributed to obtaining and analyzing of data and editing the manuscript. JMW, DSBH, KLD, and MGP contributed to implementation of experiments and analysis of data. ASK and MGP developed hypotheses for the study, supervised implementation of the study and analyses of data, and contributed to the writing and editing of the manuscript. ASK is the guarantor of this work and, as such, had full access to all the data in the study and takes responsibility for the integrity of the data and the accuracy of the data analysis.

## Supplementary Material

Supplemental data

Supplemental table 1

Supporting data values

## Figures and Tables

**Table 1:**
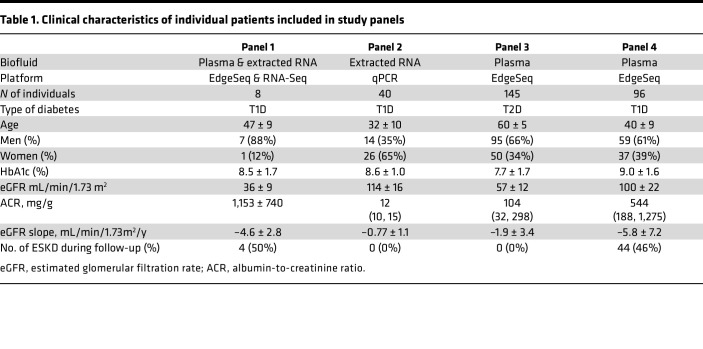
Clinical characteristics of individual patients included in study panels

**Table 2:**
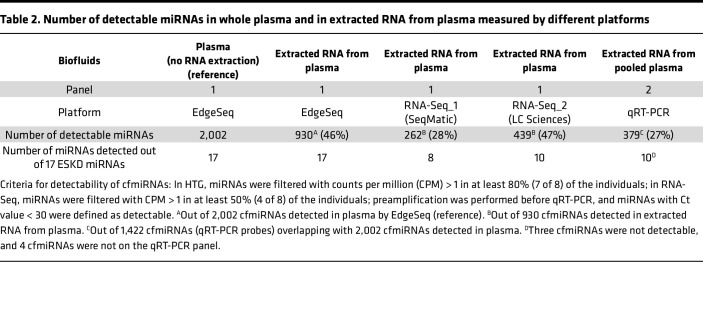
Number of detectable miRNAs in whole plasma and in extracted RNA from plasma measured by different platforms

**Table 3:**
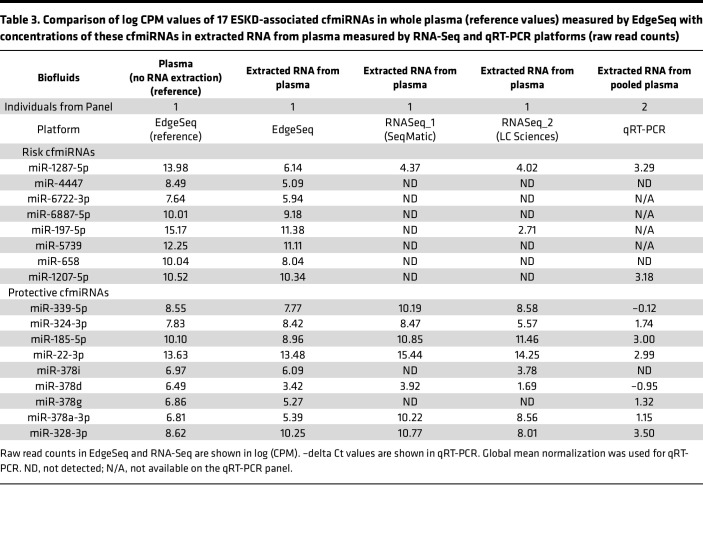
Comparison of log CPM values of 17 ESKD-associated cfmiRNAs in whole plasma (reference values) measured by EdgeSeq with concentrations of these cfmiRNAs in extracted RNA from plasma measured by RNA-Seq and qRT-PCR platforms (raw read counts)

## Version 1 06/24/2024

Electronic publication
