# Supplementary material for: Preanalytical considerations in quantifying circulating miRNAs that predict end-stage kidney disease in diabetes
Source: JCI Insight. 2024 Jun 24;9(12):e174153. doi: 10.1172/jci.insight.174153 (PMC11383361; doi:10.1172/jci.insight.174153)
Supplement: Supplemental data [file jciinsight-9-174153-s009.pdf]

SUPPLEMENTARY MATERIAL

Supplemental Table S1: Concentration of miRNAs in whole plasma and RNA extracted from plasma measured by different platforms to quantify miRNAs (Excel file).

Supplemental Table S2. Spearman correlation between raw read counts obtained using EdgeSeq platform and other platforms to measure 17 ESKD-associated cfmiRNAs in whole plasma and in RNA extracted from plasma in 8 T1D individuals.

cfmiRNA	EdgeSeq whole plasma Vs. EdgeSeq RNA	EdgeSeq whole plasma vs. RNASeq_1	EdgeSeq whole plasma vs. RNASeq_2
	r_S	r_S	r_S
miR-1287-5p	0.26	-0.39	-0.28
miR-4447	-0.20	-	-
miR-6722-3p	0.21	-	-
miR-6887-5p	-0.55	-	-
miR-197-5p	0.13	-	-0.49
miR-5739	-0.01	-	-
miR-658	0.05	-	-
miR-1207-5p	0.36	-	-
miR-339-5p	0.31	0.17	-0.57
miR-324-3p	-0.48	0.10	0.17
miR-185-5p	0.67	0.53	0.29
miR-22-3p	0.84*	0.14	0.16
miR-378i	0.57	-	-0.33
miR-378d	0.26	-0.62	0.45
miR-378g	0.19	-	-
miR-378a-3p	0.55	0.50	-0.55
miR-328-3p	0.60	0.20	0.05

r_s - Spearman correlation coefficient

***** $p<0.05$

Supplemental Table S3. Spearman correlation between storage time and raw read counts of 17 whole plasma ESKD associated cfmiRNAs.

cfmiRNA	r_s	*P*
**miR-1287-5p**	0.03	7.6E-1
**miR-4447**	-0.02	8.1E-1
**miR-6722-3p**	-0.08	3.3E-1
**miR-6887-5p**	-0.27	1.2E-3
**miR-197-5p**	0.05	5.5E-1
**miR-5739**	-0.04	6.7E-1
**miR-658**	0.24	3.9E-3
**miR-1207-5p**	0.29	4.0E-4
**miR-339-5p**	-0.03	6.8E-1
**miR-324-3p**	-0.07	3.9E-1
**miR-185-5p**	-0.11	1.9E-1
**miR-22-3p**	0.01	9.0E-1
**miR-378i**	-0.01	8.9E-1
**miR-378d**	-0.17	3.8E-2
**miR-378g**	0.02	7.7E-1
**miR-378a-3p**	0.02	7.8E-1
**miR-328-3p**	-0.03	7.2E-1

r_s - Spearman correlation coefficient

The mean ± SD of storage duration (year) was 9.3 ± 2.3 years for 145 T2D individuals.

Supplemental Table S4. Spearman correlation between raw read counts of 17 whole plasma ESKD associated cfmiRNAs and clinical characteristics in 145 T2D.

cfmiRNA	Age r_s	BMI r_s	A1c r_s	FC M/F
Risk cfmiRNA				
miR-1287-5p	0.07	0.12	0.09	0.49*
miR-4447	0.00	0.00	0.15	0.46*
miR-6722-3p	-0.04	-0.01	0.23	0.19
miR-6887-5p	0.00	0.05	0.18	0.26
miR-197-5p	0.07	0.20	0.02	0.23
miR-5739	0.01	0.09	-0.01	0.39*
miR-658	0.07	0.05	-0.04	0.39*
miR-1207-5p	0.03	-0.09	0.00	0.43*
Protective cfmiRNA				
miR-339-5p	-0.02	-0.03	-0.10	-0.23
miR-324-3p	0.07	0.02	-0.04	-0.29*
miR-185-5p	0.09	0.05	-0.04	-0.17
miR-22-3p	0.07	-0.03	-0.21	-0.24
miR-378i	-0.01	-0.03	-0.29	0.07
miR-378d	0.13	-0.10	-0.18	0.03
miR-378g	0.07	-0.02	-0.29	0.02
miR-378a-3p	0.04	-0.01	-0.24	0.02
miR-328-3p	-0.03	-0.03	0.01	-0.20

r_s - Spearman correlation coefficient

FC M/F Fold change of Men/Women

***** p<0.05 without adjustment for multiple comparisons

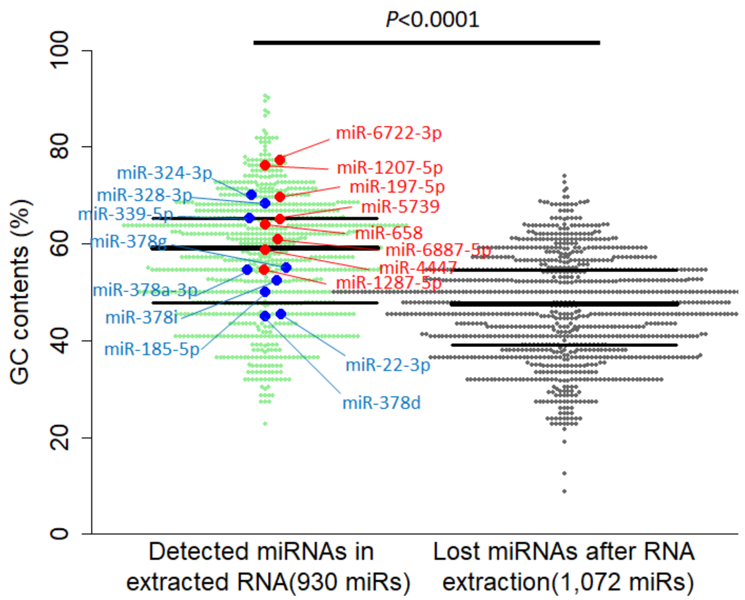

Supplemental Figure S1. Comparison of GC contents of miRNAs in extracted RNA and cfmiRNAs lost during RNA extraction. The solid lines represent the median and the thinner lines indicate lower and upper quartiles. In total, 930 cfmiRNAs (46%) were detected in extracted RNA by EdgeSeq, whereas 1,072 cfmiRNAs (54%) were lost during RNA extraction.

Supplemental Table S5. Previously reported cfmiRNAs associated with clinical characteristics.

Individuals/characteristic	cfmiRNA	method (sample type)	Up/Down	Author(s)
Healthy/Age	miR-142-5p	RNASeq and qPCR	Down	Zhang, et al (1)
	miR-29b	(RNA extracted from plasma)	Down	
	miR-106b		Down	
	miR-130b		Down	
	miR-340		Down	
	miR-92a		Up	
	miR-222		Up	
	miR-375		Up	
Healthy/Age	miR-20a	qPCR (RNA extracted from serum)	Down	Sawada et al (2)
Healthy/Age	let-7a-5p	qPCR (RNA extracted from plasma)	Down	Ameling et al (3)
	miR-126-3p		Down	
	miR-142-3p		Down	
	miR-21-5p		Down	
	miR-30b-5p		Down	
	miR-30c-5p		Down	
	miR-93-5p		Up	
Healthy/age	miR-1284	microarray + RNAseq	Up	Meder et al(4)
	miR-93-3p	(RNA extracted from whole blood)	Up	
	miR-1262		Up	
	miR-34a-5p		Up	
	miR-145-5p		Up	
Healthy/Sex	miR-145-5p	qPCR	Down	Ameling et al (3)
	miR-22-3p	(RNA extracted from plasma)	Up	
	miR-142-3p		Down	
	miR-424-5p		Up	
	miR-148a-3p		Up	
	miR-150-5p		Down	
	miR-30d-5p		Up	
Healthy/BMI	miR-106a-5p	qPCR	Up	Ameling et al (3)
	miR-122-5p	(RNA extracted from plasma)	Down	
	miR-143-3p		Up	
	miR-145-5p		Up	
	miR-148a-3p		Down	
	miR-185-5p		Up	
	miR-193b-3p		Down	
	miR-194-5p		Down	
	miR-20a-5p		Up	
	miR-215		Down	
	miR-365a-3p		Down	
	miR-505-3p		Down	

	miR-885-5p		Down	
	miR-93-5p		Up	
	miR-99a-5p		Down	
Healthy/Obesity	miR-17-5p	qPCR	Down	Heneghan et al (5)
	miR-132	(RNA extracted from whole blood)	Down	
Healthy/Obesity	miR-142-3p	qPCR	Up	Ortega et al (6)
	miR-140-5p	(RNA extracted from plasma)	Up	
	miR-222		Up	
	miR-130b		Up	
	miR-221		Up	
	miR-15a		Up	
	miR-520c-3p		Up	
	miR-423-5p		Up	
	miR-130b		Down	
Healthy/Obesity	miR-503	qPCR	Down	Pescador et al (7)
	miR-138	(RNA extracted from serum)	Down	
	miR-376a		Down	
	miR-15b		Up	
Obesity and lean	miR-21	qPCR	Down	Ghorbani (8)
		(RNA extracted from serum)		
Healthy/Obesity	miR-142-3p	qPCR	Up	Al-Rawaf et al (9)
	miR-140-5p	(RNA extracted from plasma)	Up	
	miR-222		Up	
	miR-143		Up	
	miR-130		Up	
	miR-532-5p		Down	
	miR-423-5p		Down	
	miR-520c-3p		Down	
	miR-146a		Down	
	miR-15a		Down	
Healthy/Obesity	miR-222	miRNA sequence & qPCR	Up	Cui et al (10)
	miR-486	(RNA extracted from serum)	Up	
	miR-146b		Up	
	miR-146a		Up	
	miR-20a		Up	
	miR-15b		Up	
	miR-26b'		Up	
Healthy/Obesity Brandt et al.	miR-122	qPCR (RNA extracted from serum)	Up	Brandt et al (11)
T1D individuals/HbA1c	miR-125b-5p	edgeSeq & qPCR	Up	Satake et al (12)
	miR-365a-3p		Up	
	miR-7-1-3p		Up	

miR-193a-5p	(whole plasma for edgeSeq and RNA extracted from plasma for qPCR)	Up
miR-200c-3p		Up
miR-5190		Down
miR-770-5p		Down
miR-6799-3p		Down
miR-6793-5p		Down
miR-1228-3p		Down

Supplementary references.

1. Zhang H, et al. Investigation of microRNA expression in human serum during the aging process. *J Gerontol A Biol Aci Med Sci* 2015;70(1):102-109
2. Sawada S, et al. Effect of aging and sex on circulating microRNAs in humans. *Adv Aging Res* 2014;3:152-159
3. Ameling S, et al. Associations of circulating plasma microRNAs with age, body mass index and sex in a population-based study. *BMC Med Genomics* 2015:8:61
4. Meder B, et al. Influence of the confounding factors age and sex on microRNA profiles from peripheral blood. *Clin Chem* 2014;60(9):1200-1208
5. Heneghan HM, et al. Differential miRNA expression in omental adipose tissue and in the circulation of obese patients identifies novel metabolic biomarkers. *J Clin Endocrinol Metab* 2011;96(5):E846-850.
6. Ortega FJ, et al. targeting the circulating microRNA signature of obesity. *Clin Chem* 2013;59(5):781-792
7. Pescador N, et al. Serum circulating microRNA profiling for identification of potential type 2 diabetes and obesity biomarkers. *PLoS One* 2013;8(10):e77251
8. Ghorbani S, et al. Decreased serum microRNA-21 level is associated with obesity in healthy and type 2 diabetic subjects. *Arch Physiol Biochem* 2018;124(4):300-305
9. Al-Rawaf HA. Circulating microRNAs and adipokines as markers of metabolic syndrome in adolescents with obesity. *Clin Nutr* 2019;38(5):2231-2238
10. Cui X, et al. Change in circulating microRNA profile of obese children indicates future risk of adult diabetes. *Metabolism* 2018;78:95-105
11. Brandt S, et al. Circulating levels of miR-122 and nonalcoholic fatty liver disease in pre-pubertal obese children. *Pediatric Obesity* 2018;13(3):175-182
12. Satake E, et al. Circulating miRNA profiles associated with hyperglycemia in patients with type 1 diabetes. *Diabetes* 2018;67(5):1013-1023
